# The Maximum Eigenvalue of the Brain Functional Network Adjacency Matrix: Meaning and Application in Mental Fatigue Evaluation

**DOI:** 10.3390/brainsci10020092

**Published:** 2020-02-09

**Authors:** Gang Li, Yonghua Jiang, Weidong Jiao, Wanxiu Xu, Shan Huang, Zhao Gao, Jianhua Zhang, Chengwu Wang

**Affiliations:** 1 College of Engineering, Zhejiang Normal University, Jinhua 321004, China; ligang@zjnu.cn (G.L.); jiaowd1970@zjnu.cn (W.J.); bcxwx@126.com (W.X.); huangshan@zjnu.cn (S.H.); gz@zjnu.cn (Z.G.); cwuwang@126.com (C.W.); 2 Key Laboratory of High Efficiency and Clean Mechanical Manufacture, Ministry of Education of China, School of Mechanical Engineering, Shandong University, Jinan 250061, China

**Keywords:** maximum eigenvalue, adjacency matrix, brain functional network, network characters, mental fatigue, electroencephalogram (EEG)

## Abstract

The maximum eigenvalue of the adjacency matrix (AM) has been supposed to contain rich information about the corresponding network. An experimental study focused on revealing the meaning and application of the maximum eigenvalue is missing. To this end, AM was constructed using mutual information (MI) to determine the functional connectivity with electroencephalogram (EEG) data recorded with a mental fatigue model, and then was converted into both binary and weighted brain functional network (BFN) and corresponding random networks (RNs). Both maximum eigenvalue and corresponding network characters in BFNs and RNs were considered to explore the changes during the formation of mental fatigue. The results indicated that large maximum eigenvalue means more edges in the corresponding network, along with a high degree and a short characteristic path length both in weighted and binary BFNs. Interestingly, the maximum eigenvalue of AM was always a little larger than that of the corresponding random matrix (RM), and had an obvious linearity with the sum of the AM elements, indicating that the maximum eigenvalue can be able to distinguish the network structures which have the same mean degree. What is more, the maximum eigenvalue, which increased with the deepening of mental fatigue, can become a good indicator for mental fatigue estimation.

## 1. Introduction

Brain functional network (BFN), as one type of the complex networks, is a demonstration of the temporal and topological correlations among different brain regions in the processes of brain neural activities [1]. It should ignore some technical details when constructing BFNs, such as the size, location, and shape of the network node, and the physical distance and geometric shapes of the connective edge. The mapping techniques of the BFNs is mainly based on the data of electroencephalogram (EEG) [2], magnetoencephalogram (MEG) [3], positron emission tomography (PET) [4], functional magnetic resonance imaging (fMRI) [5], near infrared reflectance spectroscopy (NIRS) [6], etc. For the establishment of BFNs, some methods of measuring the functional connectivity between different brain regions have been used, such as cross correlation, partial correlation, Pearson correlation, coherence, mutual information (MI), synchronization likelihood, etc. Commonly explored BFN characters involve the degree, centrality, characteristic path length, clustering coefficient, and efficiency [7]. These BFN characters have been applied to explore the changes in brain functions, such as the normal subjects that in different states [8], that with different education levels [9], that in different age stages [10], and that at different sleep stages [11], as well as the subjects that suffering from mental disorders [12]. BFN has become a widely-used method to study the brain sciences.

In addition, some other researchers concentrate on the adjacency matrix (AM) of BFN [13,14,15,16,17]. They believe that the topological structure of BFN can be completely depicted by the corresponding AM [13]. The eigenvalues, defined as the spectra of the AM, are supposed to contain a wealth of information in regard to network features [14]. Existing works indicate that the spectra of large-scale networks turn out to obey characteristic power-law distribution on the basis of the spectral analysis of the networks [14]. It has been also reported that a bigger maximum eigenvalue of AMs means more numbers of functional connectivities (network edges) in the corresponding BFNs [15], resulting in a larger degree and clustering coefficient and smaller characteristic path length [16,17]. However, the meaning of maximum eigenvalue in BFN analysis and the relationship between maximum eigenvalue and network characters in different network types with the same mean degree are still not explicitly revealed by the previous studies.

Mental fatigue, referring to a state of reduced mental alertness and impaired performances, is often associated with worsening performance on cognitive tasks, such as increased propensities for errors and slowed reaction times. Mental fatigue has become one of the most significant causes of traffic accidents [18]. An increasing number of researchers are motivated to develop an efficient mental fatigue evaluation indicator to avoid the mental fatigue related traffic accidents. The EEG data are emerged in mental fatigue related studies [19,20], focusing on the changes of EEG frequency components’ energy of each electrode in the early stages and failing to characterize the interactions between different EEG electrodes [21]. Recently, more studies evaluate the mental fatigue with the network characters of BFN [2,13,22,23].

In the present study, we attempt to study the meaning of maximum eigenvalue in BFN analysis and the application of the maximum eigenvalue in mental fatigue detection with the EEG data recorded with a mental fatigue model. To this end, the relationship between the maximum eigenvalue and the sum of the adjacency matrix elements, and the changing regularities of maximum eigenvalue and network characters (degree and characteristic path length) in weighted/binary BFNs and corresponding weighted/binary RNs were analyzed during the formation of mental fatigue. We also tried to estimate mental fatigue using maximum eigenvalue and network characters. Functional connectivities between any two brain regions were determined with MI in different EEG frequency bands. These functional connectivities were used to establish the AMs, which were then used to extract the BFNs with a threshold for the further analysis.

## 2. Materials and Methods

### 2.1. Participants

Twenty healthy male subjects (females were excluded to eliminate the effects of sex differences on the results) of engineering postgraduate students were recruited. They were all right-handed, aged from 23 to 26 (24.5 ± 1.5). Their mean Body Mass Index was 20.7 ± 1.8 kg/m^2^. All participants were asked to report to have a regular routine, have normal or rectified eyesight, have no brain diseases, and not stay up late and drink alcohol and take drugs in the previous week before the EEG data acquisition (EEG DAQ). Smoking, coffee, and strong tea were forbidden in eight hours before EEG DAQ for all participants. In addition, they must wash their hair in two hours before EEG DAQ. All subjects have signed their informed consent for this study. The Shandong University Ethical Committee approved the study.

### 2.2. EEG Data Acquisition and Preprocessing

**Figure 1:**
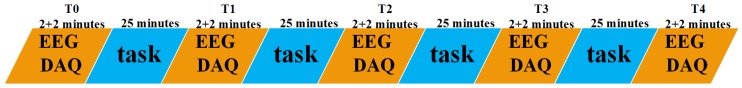
Electroencephalogram data acquisition (EEG DAQ) procedures.

EEG DAQs were implemented by a digital EEG apparatus (SYMTOP NT9200) among the participants at the following 19 positions of the 10–20 systems: Fp1, Fp2, F3, F4, C3, C4, P3, P4, O1, O2, F7, F8, T3, T4, T5, T6, Fz, Cz, and Pz (Fpz is the grounding electrode, A1 and A2 are the reference electrodes). The sample frequency was 1000 Hz. Band-pass filter of 0.5–70 Hz was carried out during EEG DAQs. Electrode impedance was controlled below 5000 Ω. A mental fatigue model was designed to induce mental fatigue among the participants by doing a task of 200 different mental arithmetic math problems. The mental arithmetic math problem, which was limited to be completed in 30 seconds determined by preceding pretests in a suitable difficulty level, was designed as a double digits between sixty and ninety plus another double digits between sixty and ninety then multiplied by a single digit between six and nine. The whole task was divided equally into four task segments (contain 25 different mental arithmetic math problems) and was accomplished from 7 pm to 9 pm. EEG data were recorded before and after every task segment. Therefore, five times of EEG DAQs, named as T0, T1, T2, T3, and T4 shown in Figure 1, were implemented during the whole task for each participant. Additionally, 2-minute EEG recordings for both resting state and task state were considered for every EEG DAQ. Resting state means the participants should be relaxed, awake, and closing the eyes, and required to focus the attention on the breath and avoid thinking. While the task state means the participants should do a mental arithmetic task, a three-digit number subtracts a single digit continuously (kept the same for all EEG DAQs). All tasks were displayed on a computer screen and come out one by one. All EEG DAQs were carried out in a sound attenuated, temperature, humidity, and light controlled laboratory.

EEG signals from 18 participants were taken into account for following analysis, for the other two were excluded because of the larger fluctuation of the head. Ten pieces of 5-second artifact-free contiguous EEG data (eye blinks, slow eye movements, and electrocardiogram artifacts were eliminated by Fast ICA [24], and baseline drift were removed by baseline correction.) were extracted from 2-minute EEG data for each state by EEGLAB. That is, there were 100 pieces of EEG data obtained for the whole analysis. These pieces of EEG data were then down sampled from 1000 Hz to 256 Hz using MATLAB. After Fast Fourier Transform (FFT, embedded in MATLAB) on the EEG data to distinguish the traditional EEG rhythms (2–4 Hz for delta, 4–8 Hz for theta, 8–10 Hz for alpha1, 10–13 Hz for alpha2, 13–30 Hz for beta), the MIs between all pairs of EEG channels were calculated by a MATLAB program written by Moddemeijer [25] to determine the functional connectivity, resulting in undirected 19 × 19 AMs.

### 2.3. Computation of the Maximum Eigenvalue and Network Characters

In this part, all the computations were completed with MATLAB. Both weighted and binary AMs and their corresponding random matrices (RMs) were taken into consideration. An AM is a means of representing which nodes of a network are adjacent to which other nodes. The weighted AM was gained by choosing a threshold *T* and setting the MI values to zero when their MI values were smaller than *T*, but keeping the other values unchanged in AM. The binary AM was defined on the basis of the weighted AM and setting the MI values to 1 when their MI values were greater than *T*. The RM, corresponding to AM (including weighted and binary AM), was obtained by randomly disrupting the order of MI values, still keeping the matrix symmetric and zeroes on its main diagonal. The weighted/binary BFNs and weighted/binary random networks (RNs) were determined by the corresponding AMs and RMs with the given threshold *T*. However, there is no unique way to choose *T*, thus a series of values of *T*, 0.15 ≤ *T* ≤ 0.35 with increments of 0.01, were applied to repeat all analysis under every given value of *T*.

The maximum eigenvalue and the network characters, degree and characteristic path length (computed with BCT toolbox embedded in MATLAB), were defined in Equations (1)–(7), respectively. In the equations, *N* is the set of all nodes in the network, *n* is the number of nodes, *a_ij_* is the connection between node *i* and node *j* (*a_ij_* = 1 when link exists; *a_ij_* = 0 otherwise), *w_ij_* is the weight between node *i* and node *j*, *i* and *j* belong to *N*. Where, in Equation (1) and Equation (2), **v** is the eigenvector corresponding to eigenvalue *λ*, **A** is the AM, and *λ*_max_ is the maximum eigenvalue. In Equation (3) and Equation (4), *K* and *K^w^* are the binary and weighted degree, respectively. In Equation (5), Equation (6), and Equation (7), *l_ij_* and $l_{ij}^{w}$ are the binary and weighted shortest path length extracted from all possible path lengths between node *i* and node *j*, respectively, and *L* and *L^w^* are binary and weighted characteristic path length, respectively. Additionally, the shortest path length was calculated by Dijkstra algorithm [26].

(1) Av=λv

(2) $$\lambda_{\max}=\max\left\{\left|\lambda_{1}\right|,\left|\lambda_{2}\right|\cdots \left|\lambda_{n}\right|\right\}$$

(3) $$K=\frac{1}{n}\sum a_{ij}$$

(4) $$K^{w}=\frac{1}{n}\sum w_{ij}$$

(5) $$L=\frac{1}{n(n-1)}\sum_{i\neq j\in N}l_{ij}$$

(6) $$L^{w}=\frac{1}{n(n-1)}\sum_{i\neq j\in N}l_{{}_{ij}}^{w}$$

(7) $$l_{ij}^{w}=\min(\frac{1}{w_{ik}}+\frac{1}{w_{kf}}+\cdots +\frac{1}{w_{mn}}+\frac{1}{w_{nj}})$$

### 2.4. Statistical Analysis

One-way ANOVA was implemented to identify significant statistical differences among the 5 time points (T0, T1, T2, T3, and T4). This ANOVA analysis was performed on the mean maximum eigenvalue of delta, theta, alpha1, alpha2, and beta using the program embedded in MATLAB. Results are demonstrated as mean ± SD. Significant level is reported at *p* < 0.05.

## 3. Results

**Figure 2:**
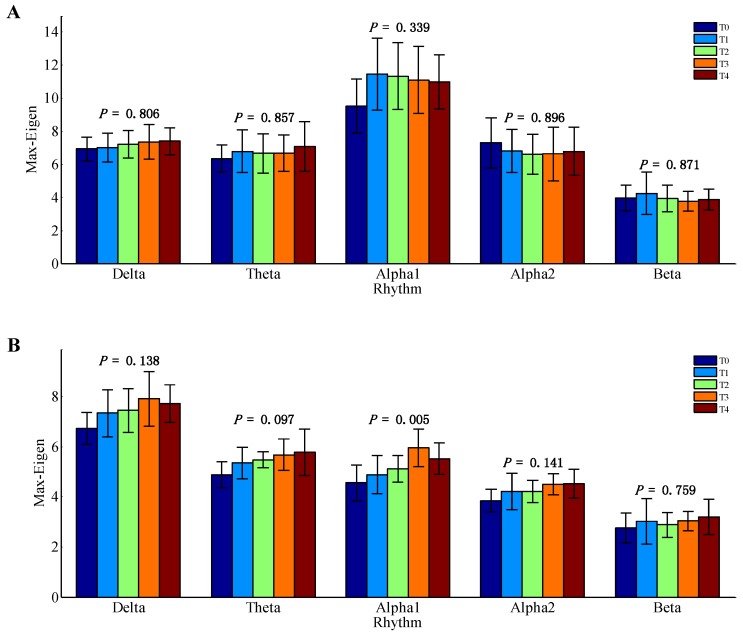
Mean maximum eigenvalue (Max-Eigen) of the adjacency matrix (AMs) for all EEG rhythms among T0, T1, T2, T3, and T4 during the formation of mental fatigue, with no threshold (*T* = 0). The bar in the figures means the standard deviation across the subjects. The *p*-values of the one-way ANOVA are also given in the figures. (**A**) Resting state. (**B**) Task state.

Results of mean maximum eigenvalue of the AMs for delta, theta, alpha1, alpha2, and beta EEG rhythms during the formation of mental fatigue are shown in Figure 2. Only the data of alpha1 rhythm at task state were emerged according to the results of one-way ANOVA (*p* = 0.005). Whereas, the other nine groups of EEG data have no significant differences (*p* > 0.05). Therefore, the EEG data of alpha1 rhythm at task state were considered for further analysis.

**Figure 3:**
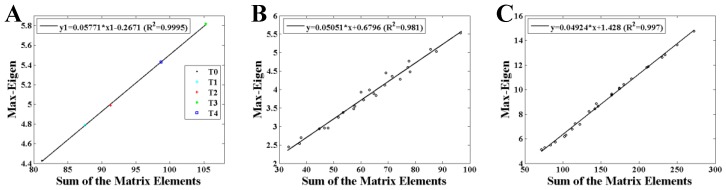
Relationship between the maximum eigenvalue and the sum of the AMs elements for alpha1 rhythm at task state. (**A**) Results of the AMs with no threshold (*T* = 0). (**B**) Results of the weighted AMs with the thresholds of *T* = 0.15, 0.20, 0.25, 0.30, 0.35. (**C**) Results of the binary AMs with the threshold of *T* = 0.15, 0.20, 0.25, 0.30, 0.35.

**Figure 4:**
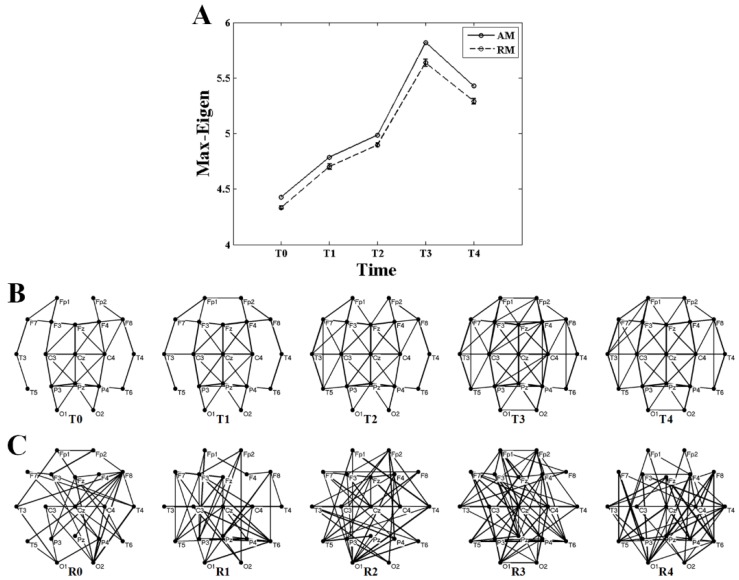
Differences of the maximum eigenvalue (Max-Eigen) between AM and random matrix (RM). (**A**) Results of the maximum eigenvalue of AM and RM with no threshold (*T* = 0) for alpha1 rhythm at task state. The results of the RM were acquired from averaging two-hundred RMs. The bars indicate the standard error of mean. (**B**) Networks converted by the AMs using a threshold of *T* = 0.35 for T0, T1, T2, T3, and T4. (**C**) Networks converted by the RMs using a threshold of *T* = 0.35, corresponding to the networks of the AMs. In (**B**) and (**C**), if the mutual information (MI) value between 2 electrodes is above the threshold, an edge is drawn between the 2 vertices, otherwise not. The boldness of the line indicated the size of the MI value. Networks were drawn by Pajek.

Figure 3 and Figure 4 show the statistical results of the maximum eigenvalue. As shown in Figure 3, an obvious linearity between the maximum eigenvalue and the sum of the AM elements (MI values) is obtained both in weighted and binary AMs. Figure 4 shows the differences of the maximum eigenvalue between AMs and corresponding RMs. It is clearly demonstrated that the maximum eigenvalue of RMs is always a little smaller than that of AMs, but they have the same variation trend (Figure 4A). Comparing the networks converted by the AMs and RMs using a threshold of *T* = 0.35 shown in Figure 4B,C, it is shown that BFNs have more regular structures than RNs.

**Figure 5:**
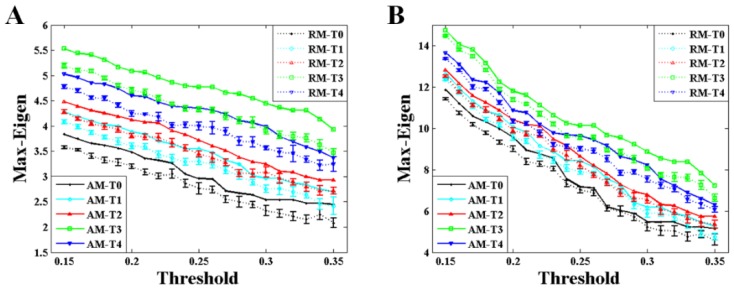
Results of the maximum eigenvalue (Max-Eigen) obtained from AM and RM for alpha1 rhythm at task state. The results of the RM were acquired from two-hundred RMs. Error bars correspond to standard error of the mean. (**A**) Results of the weighted matrices. (**B**) Results of the binary matrices.

Figure 5 shows the comparison of the maximum eigenvalue between AM and RM with different values of threshold during the formation of mental fatigue. The variation trend of the maximum eigenvalue, strictly increasing before T3 and having a small drop but larger than T2 at the time of T4, almost keeps the same during the whole given threshold. This trend is applicable in the results of both weighted and binary AM/RM as shown in Figure 5A,B. What is more, the maximum eigenvalue obtained from RM is smaller than that from AM all the time.

**Figure 6:**
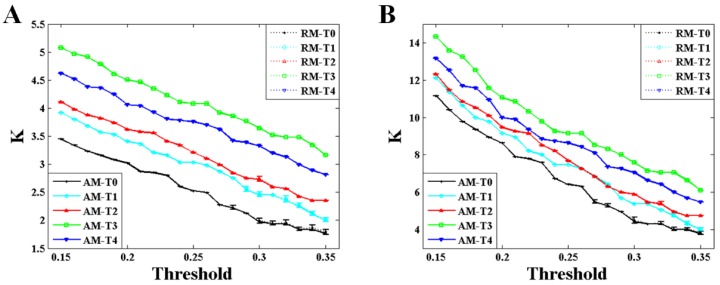
Results of the degree obtained from AM and RM for alpha1 rhythm at task state. The results of the RM were acquired from two-hundred RMs. Error bars correspond to standard error of the mean. (**A**) Results of the weighted networks. (**B**) Results of the binary networks.

**Figure 7:**
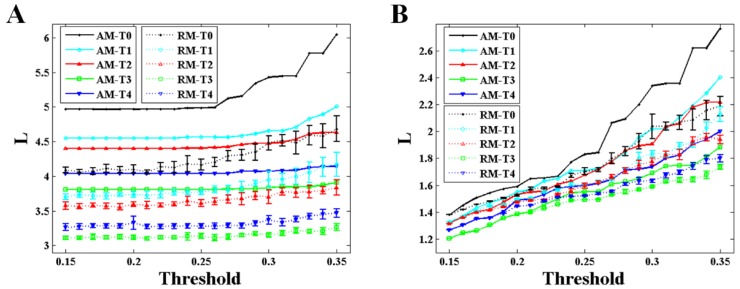
Results of the characteristic path length obtained from AM and RM for alpha1 rhythm at task state. The results of the RM were acquired from two-hundred RMs. Error bars correspond to standard error of the mean. (**A**) Results of the weighted networks. (**B**) Results of the binary networks.

As depicted in Figure 6 and Figure 7, the variation trends of degree and characteristic path length show a significant consistency with that of the maximum eigenvalue during the formation of mental fatigue. With the increase of the maximum eigenvalue, the degree increases and the characteristic path length decreases. In addition, the strong correlation between the maximum eigenvalue and the network characters keeps the same in RNs, showing in the results of the dashed lines. What is more, comparing the results of the network characters between BFNs and RNs, it shows that the results of characteristic path length are similar with the results of the maximum eigenvalue. No differences in degree can be observed in these two types network.

## 4. Discussion

In the current study, we attempted to explore the meaning of maximum eigenvalue in BFN analysis, and estimate the mental fatigue using complex network theories [23,27,28]. Both the maximum eigenvalue and the network characters of degree and characteristic path length were calculated to discuss the mental fatigue process. As depicted in Figure 2, significant differences of the maximum eigenvalue among the time of T0, T1, T2, T3, and T4 were found only in alpha1 (8–10 Hz) band at task state. Thus, only these EEG data were considered for further analysis. What is more, the same frequency band of alpha1 rhythm was also explored in the study of functional cortical connectivity analysis of mental fatigue [23].

The maximum eigenvalue of the AM showed a significant consistency with the network characters, and can be used to predict the network characters and distinguish the network structures which have the same mean degree. As depicted in Figure 5, Figure 6 and Figure 7, a large maximum eigenvalue of the AM means that the corresponding network had a large degree and a short characteristic path length, seen both in weighted and binary networks. As it is known, the network characters are directly determined by the BFN converted from the AM, indicating the concordance between the maximum eigenvalue of the AM and the corresponding network characters [29]. To reveal the further correlation of this unique consistency, some interesting results had been obtained. As shown in Figure 3, the maximum eigenvalue has a good linearity with the sum of the AM elements shown both in weighted and binary AM. An inference can be made from these results that the whole MI values of the AM are larger if the maximum eigenvalue is higher. That is, the network is denser when the corresponding AM has a larger maximum eigenvalue, perfectly supported by the results shown in Figure 4A,B. A similarity has been reported by Dorogovtsev et al. that the size of a network (number of network edges) determines the largest eigenvalue in its spectrum [15]. The increase in degree can naturally make the characteristic path length short. These can be obviously obtained by the definitions of the characteristic path length. It means that a denser network obviously has a larger degree and a shorter characteristic path length. What is more, the maximum eigenvalue can completely distinguish the BFN and its corresponding RN which have the same mean degree, that the maximum eigenvalue obtained from RMs is always smaller than that from AMs as shown in Figure 4 and Figure 5. From the above, it can be concluded that the maximum eigenvalue shows great significance in network analysis.

The maximum eigenvalue increases at successive times, existing a small discordance at time T4, probably due to the adjustments of physiological rhythm. Similar phenomena, which the metrics are not monotonically increased with the increasing level of mental fatigue, are also observed in previous works [30,31]. The Regular change of the maximum eigenvalue implied that the maximum eigenvalue can be considered as a criterion to estimate the mental fatigue. In a study of eigenvalue with fMRI data [14], the maximum eigenvalue decreases with the increasing of the threshold, and the maximum eigenvalue is larger in second sensorimotor task than that in first sensorimotor task during the whole threshold. The results in that study [14] are very compatible with ours shown in Figure 5. Along with the increase in maximum eigenvalue, significant increase in degree and decrease in characteristic path length can be also observed both in weighted and binary BFNs, suggesting that network characters can also be used to estimate the mental fatigue. In Figure 4B, it shows that the network has an increasing numbers of functional connectivities (network edges) during successive times of the experiment [23,27,28]. This increase in functional connectivities can naturally result in the increase in degree and the decrease in characteristic path length. In controlled experiments using a period of 36 hours of sleep deprivation as a means to induce fatigue [27], it has been found that the results of degree and characteristic path length show highly coincident with ours.

Regular changes in maximum eigenvalue and network characters indicated that these metrics can also be used to explain the mental fatigue. As shown in Figure 4B, more functional connectivities were activated basing on the prior BFN with the deepening of the mental fatigue for the successful performance of sustained mental arithmetic subtraction task, revealing better synchronization of alpha1 rhythm between different brain regions [27]. The increasing regularity in functional connectivities is compatible with the two fundamental principles of the functional organization during cerebral information processing: functional separation and integration [32], which is to satisfy the brain for the fast real-time response to internal and external environment changes [33]. At incipient stage (no mental fatigue), completing an attention task just needs a smaller functional connectivities. In order to finish the same task at mental fatigue stage, the brain should activate more functional connectivities. In a word, the BFN should turn to have higher maximum eigenvalue and degree and shorter characteristic path length to meet the deepening mental fatigue. By the way, no significant differences were observed for the weighted and binary BFNs to demonstrate their network features.

Our current study still has some limitations. On the one hand, we concluded that significant alterations in maximum eigenvalue, degree and characteristic path length were found only in the alpha band at task state during the formation of mental fatigue. We fail to provide the physiological background. Much more attention should be paid to this field. On the other hand, both the number of EEG electrodes and sample size are small in this study. More experimental studies should be conducted to provide sufficient evidence to support the conclusions of this study. 

## 5. Conclusions

In this study, a group of strictly controlled experiments was conducted to study the meaning and application of the maximum eigenvalue with a mental fatigue model. The results showed a strong correlation between the maximum eigenvalue and the network characters, revealing a meaningful conclusion that the maximum eigenvalue of the AM had a key position in studying the complex network. If the AM had a big maximum eigenvalue, the corresponding network would have a large degree and a short characteristic path length both in weighted and binary networks. Interestingly, the maximum eigenvalue of BFN was always larger than that of the corresponding RN, and had an obvious linearity with the sum of the AM elements, indicating that the maximum eigenvalue can be used to distinguish the network types which have the same mean degree. What is more, the maximum eigenvalue can be used to evaluate the mental fatigue, as well as the network characters. With the deepening of mental fatigue, more functional connectivities between different brain regions were activated basing on the prior BFN, along with the maximum eigenvalue and degree increased, and the characteristic path length decreased.
